# Ammonia-Assimilating Bacteria Promote Wheat (*Triticum aestivum*) Growth and Nitrogen Utilization

**DOI:** 10.3390/microorganisms13010043

**Published:** 2024-12-30

**Authors:** Yuqian Gao, Qi Zhang, Yuannan Chen, Yanqing Yang, Chenxiao Zhou, Jiayang Yu, Yanan Li, Liyou Qiu

**Affiliations:** Key Laboratory of Enzyme Engineering of Agricultural Microbiology, Ministry of Agriculture and Rural Affairs, College of Life Sciences, Henan Agricultural University, Zhengzhou 450046, China; yqgao2005@henau.edu.cn (Y.G.); yangyanqing1203@163.com (Y.Y.); 15836771621@163.com (J.Y.)

**Keywords:** ammonia-assimilating bacterium, ammonium immobilization, *Enterobacter*, wheat, ammonia nitrogen accumulation, total nitrogen accumulation

## Abstract

Nitrogen fertilizers in agriculture often suffer losses. Ammonia-assimilating bacteria can immobilize ammonia and reduce these losses, but they have not been used in agriculture. This study identified an ammonia-assimilating strain, *Enterobacter* sp. B12, which assimilated ammonia via the glutamate dehydrogenase (GDH) pathway at low levels (5 mM) and the glutamine synthetase (GS)-glutamine-2-oxoglutarate aminotransferase (GOGAT) pathway at high levels (10 mM). Inoculating wheat with B12 increased seedling dry weight, nitrogen accumulation, rhizosphere soil nitrogen content, and root enzyme activities, including GDH, superoxide dismutase (SOD), catalase (CAT), and peroxidase (POD), under both conditions. However, root GS, GOGAT enzyme activities, and ammonia assimilation-related gene expressions were lower than the controls. The results suggest that the ammonia-assimilating bacterium promotes wheat growth, nitrogen accumulation, and soil nitrogen immobilization by establishing an ammonia and amino acid exchange with roots and enhancing root antioxidant capacity, making it a potential plant growth-promoting rhizobacteria (PGPR).

## 1. Introduction

To meet the growing population’s demand for agricultural production, the global use of fertilizers, especially nitrogen, has been increasing rapidly. In 2020, the global application of nitrogen fertilizer was 112,364.0 thousand metric tons; among them, ammonia nitrogen fertilizers are the main form, with a small amount of nitrate nitrogen [1]. Half of the nitrogen fertilizer is applied to the world’s three major cereal crops: rice (16%), wheat (18%), and maize (16%). Unfortunately, around 50% of N fertilizer is lost through NH_3_ volatilization, NO_3_^−^ leach and runoff, and emission from denitrification (producing NO, N_2_O, and N_2_ gases), as well as immobilized by other organisms or soils [2,3]. For example, only 48% of the 21.6 Tg (teragram) of N applied annually in global wheat is utilized, with losses of 1–21% by NH_3_ volatilization, 2.3–43.5% by NO_3_^−^ leaching, and 0.24–1.21% by N_2_O emission [2]. Lost N not only limits crop yield increases and raises costs but also contributes to atmospheric greenhouse gas emissions, soil acidification, groundwater pollution, and surface water eutrophication [4]. In addition, excessive use of ammonia nitrogen fertilizers has resulted in high levels of ammonia in the soil, which is toxic to plants and causes growth inhibition, leaf chlorosis, and oxidative stress [5,6].

Enhanced efficiency fertilizers (EEFs) can effectively reduce nitrogen losses by utilizing coatings, urease inhibitors, and nitrification inhibitors to stabilize, slow-release, and control the release of nitrogen. Membrane-/film-based mulching methods also achieve NH_3_ mitigation via the barrier effect [7]. Another alternative to reduce nitrogen loss is the use of biofertilizers. *Trichoderma viride* biofertilizer lowers the pH of alkaline soils and increases the abundance of ammonium-oxidizing archaea (AOA) and ammonium-oxidizing bacteria (AOB), which promote the conversion of NH_4_^+^ to NO_3_^−^, thus reducing NH_3_ volatilization [8]. *Bacillus* biofertilizers decrease the conversion of fertilizer nitrogen to NH_4_^+^-N and simultaneously accelerate NH_4_^+^-N nitrification, reducing the NH_4_^+^-N content remaining in alkaline soil and consequently alleviating the ammonia volatilization [9,10]. However, ammonia-assimilating bacteria biofertilizers have not yet been employed.

Ammonia-assimilating bacteria consume ammonia by assimilation rather than nitrification [11] and are widely distributed in ammonia-rich environments such as rumen and compost [12,13,14]. Ammonia-assimilating bacteria assimilate ammonia in the same way as other organisms, including the glutamate dehydrogenase (GDH) pathway and the glutamine synthetase (GS)–glutamine-2-oxoglutarate aminotransferase (GOGAT, also recognized as glutamate synthase) pathway. Bacterial ammonia assimilation has been more intensively studied in *Escherichia coli*, where the GDH pathway does not require adenosine triphosphate (ATP), whereas the GS-GOGAT pathway does. As a result of this energy exchange, GS exhibits a high affinity for ammonia (*K*_m_~0.1 mM), while GDH has a low affinity for ammonia (*K*_m_~1.5 mM) [15]. Therefore, in high ammonium and low carbon/energy media, ammonia is assimilated by the GDH pathway, and in low ammonium and high carbon/energy media, ammonia is assimilated by the GS-GOGAT pathway [16].

Ammonium (NH_4_^+^ + NH_3_) is the optimal nitrogen source for *E. coli*, resulting in the most rapid growth. At pH 7.4, NH_3_ comprises about 2% of the total ammonium and crosses the cell membrane via unmediated diffusion. When the pH drops to 5.5, NH_3_ constitutes only about 0.02% of the total ammonium. At both pH 7.4 and 5.5, NH_4_^+^ constitutes the majority of the total ammonium and enters the cell only through the expression and function of the ammonium transporter protein AmtB. Under low-ammonium conditions, the *E. coli* Δ*amtB* strain grew very slowly, whereas under high-ammonium conditions, the Δ*amtB* strain grew similarly to the WT strain [17].

In higher plants, ammonia assimilation is dominated by the GS-GOGAT pathway, and GS is the key enzyme localized in different locations, GS1 (cytosolic GS) being cytosolic and GS2 (plastidic/chloroplast GS) plastidic. There are two types of GOGAT in higher plants: Fd-GOGAT, which uses ferredoxin as an electron donor, and NAD-GOGAT, which uses NADH (nicotinamide adenine dinucleotide) as an electron donor [18]. *GS1;1* and *GS1;4* genes are inducibly expressed in *Arabidopsis* leaves under nitrogen starvation [19], whereas GS2 is mainly affected by light. The GDH pathway has been suggested to be used as an alternate route for NH_4_^+^ assimilation in higher plants under abiotic stress [20,21].

The uptake of NH_4_^+^ by the plant roots is regulated by the NH_4_^+^ concentration in the environment. When environmental NH_4_^+^ is below 1 mM, the roots take up with the high-affinity transport system, such as AtAMT1;1 and AtAMT1;3 in *Arabidopsis* [22]. The low-affinity transport system is activated when NH_4_^+^ exceeds 1 mM [23]. No specific transport proteins have been identified for the root low-affinity transport system.

Wheat (*Triticum aestivum*) is the second largest grain crop worldwide, with urea [CO(NH_2_)_2_] and ammonium sulfate [(NH_4_)_2_SO_4_] being the most commonly used base fertilizers for wheat cultivation [24,25]. It contains three cytosolic GS1, TaGS1.1-1.3, one plastidic TaGS2, one NAD-GOGAT-type TaNADH-GOGAT1, and one Fd-GOGAT-type TaFd-GOGAT1. Ammonia strongly induced the expression of TaGS1.1 and TaGS1.3 in wheat roots, while TaGS1.2 was mainly located in the surrounding vessels of xylem [26]. During the grain-filling stage, TaGS1.3 was stably expressed, and the activity of TaNADH-GOGAT1 is much higher than that of TaFd-GOGAT1. Overexpression of TaNADH-GOGAT1-3B increased wheat yield [18]. However, wheat is very sensitive to ammonia toxicity. High ammonia suppresses wheat root hair formation and reduces seed germination [27]. The alleviation of ammonium toxicity in plants still lacks effective strategies [28].

In a previous study, an ammonia-assimilating bacterium strain was isolated from the compost of oyster mushrooms (*Pleurotus ostreatus*) and numbered B12. This strain is resistant to high ammonia and high temperatures. When inoculated into the substrate for oyster mushroom cultivation, it can promote composting, reduce ammonia volatilization during the compost process, increase the nitrogen content of the compost, and enhance mushroom yield [29]. In this study, the ammonia assimilation characteristics and classification of the isolated ammonia-assimilating bacterium B12 were identified. Inoculating potted wheat with this strain promoted wheat growth, nitrogen utilization, nitrogen immobilization in the rhizosphere soil, and improved root antioxidant enzyme activity. Additionally, it modulated the expression of ammonia assimilation-related genes and amino acid transporters in the wheat roots.

## 2. Materials and Methods

### 2.1. Strains, Plants, and Plasmids

Ammonia-assimilating bacterium strain B12 was isolated from the compost of oyster mushroom [29]. Wheat (*Triticum aestivum*) seeds, Bainong 207, purchased from Qiule Seeds (Zhengzhou, China). pCas-SSB and pTargetF stored in our lab [30]. pDONR207 was from MiaoLing Plasmid Platform (Wuhan, China). pCas-SSB is derived from the plasmid pCas [31], which expresses Cas9 and λ-Red by replacing λ-Red with a single-stranded DNA binding protein (SSB) from *E. coli*. pTargetF expresses sgRNA [31]. pDONR207 contains the chloramphenicol resistance gene expression cassette.

### 2.2. Identification of Ammonia Assimilating Strain

The 16S rRNA gene sequence of the strain B12 was compared with that of the type strain from the EzBioCloud database (http://www.ezbiocloud.net/, accessed on 12 January 2024) [32]. Phylogenetic trees based on 16S rRNA gene sequences were generated by the neighbor-joining method using the software package MEGA (molecular evolutionary genetics analysis) version 6 [33].

### 2.3. Determination of Ammonia Assimilation Rate

Ammonia–nitrogen medium was used to determine the rate of ammonia assimilation by ammonia-assimilating bacteria. The composition per liter of ammonia–nitrogen medium was as follows: glucose—3.75 g, NH_4_Cl—0.50 g, K_2_HPO_4_—1.00 g, K_2_HPO_4_—0.30 g, MgSO_4_-7H_2_O—0.05 g, FeSO_4_-7H_2_O—0.01 g, MnSO_4_-4H_2_O—0.01 g, and NaCl—5.00 g; and the pH adjusted to 7.0. The NH_4_Cl concentrations in the ammonia–nitrogen medium were set to 10 mM, 40 mM, 65 mM, and 100 mM to grow the ammonia-assimilating strain by shaking flask incubation at 30 °C and 220 rpm for 24 h. After centrifugation at 10,000 rpm for 2 min, the ammonia nitrogen concentration in the supernatant was determined by using the indophenol blue (IPB) method with *o*-phenylphenol (OPP) [34]. The ammonia assimilation rate was calculated according to the following formula [35]:

$$\mathrm{AAR}\;(\%)=(1-\frac{C_{1}}{C_{0}})\times 100$$

where AAR is the ammonia assimilation rate, *C*_0_ is the initial ammonia concentration (%), and *C*_1_ is the residual ammonia concentration (%).

### 2.4. Determination of Nitrite and Nitric Acid Content

Determination of nitrite and nitric acid in the bacterial supernatants was performed by flow injection analysis [36].

### 2.5. Bacterial Growth Curve

Bacterial strains were incubated at 30 °C and 220 rpm in ammonia–nitrogen medium with 10 mM and 30 mM NH_4_Cl or glutamine as the nitrogen source, respectively, and the OD_600_ was measured every 4 h to plot growth curves [35].

### 2.6. DNA Extraction, 16S rRNA Gene and Genome Sequencing

Bacterial cells cultured in ammonia–nitrogen medium shake flasks for 12 h were collected, and genomic DNA was extracted using the FastPure Bacteria DNA Isolation Mini Kit (Vazyme, Nanjing, China). The 16S rRNA gene was selectively amplified from the genomic DNA by PCR, as described [37], with the universal primers P1 and P6 (Table S1) [38].

The random fragmentation, library construction, and sequencing of bacterial genomic DNA were conducted by Sangon Biotech (Shanghai, China) using the Illumina NovaSeq 6000 platform. After sequencing, raw reads were filtered to obtain clean reads by Trimmomatic (v0.36) [39]. The genome assembly was performed using SPAdes (v3.15) [40]. Gaps in the assembled contigs were filled using GapFiller [41], and the sequences were corrected using PrInSeS-G [42]. Gene element prediction for the assembly was conducted with Prokka [43]. RepeatModeler was employed for de novo prediction of repetitive sequences, and RepeatMasker was used to identify the locations and frequencies of various types of repetitive sequences in the genome segments. The whole genome was annotated using NCBI Blast+ by searching against the databases including the Conserved Domain Database (CDD), euKaryotic Ortholog Groups (KOG), Clusters of Orthologous Groups of proteins (COG), NCBI nonredundant protein sequence (NR), NCBI nucleotide sequence (NT), SwissProt (a manually annotated and reviewed protein sequence database), TrEMBL, Gene Ontology (GO), and Kyoto Encyclopedia of Genes and Genomes (KEGG).

### 2.7. Quantitative PCR

Total RNA from ammonia-assimilating bacterium B12 and wheat roots was extracted using TRIeasy™ Total RNA Extraction Reagent (Yeasen, Shanghai, China). Hifair^®^ Advance Fast One-step RT-gDNA Digestion SuperMix for qPCR (Yeasen, Shanghai, China) was used to remove genomic DNA contamination and synthesize cDNA. The ammonia assimilation-related gene expression in B12 and wheat roots was analyzed by quantitative PCR with the cDNA used as a template, and the primers used were listed in Table S2. The reference genes for B12 and wheat were *rpoB* and *TaActin*. The relative gene expression level was calculated using the 2^−ΔΔCT^ method [44].

### 2.8. Construction of Ammonia Assimilation-Related Gene Deletion Mutant

The pCas-SSB/pTrgetF homologous recombination gene editing method [30] was used to construct strain B12 ammonia assimilation-related gene deletion mutants. The construction of pTargetF-*gdhA*, pTargetF-*glnA*, pTargetF-*gltD*, and pTargetF-*amtB* was performed using the primer site mutation of pTargetF using the Fast Mutagenesis System kit (TransGen Biotech, Beijing, China) following the manufacturer’s protocol. The primers used were listed in Table S1. The homologous recombinant donor DNA fragment consists of upstream and downstream homology arms and a chloramphenicol resistance gene expression cassette. The upstream and downstream homology arms were amplified by PCR using the B12 genome as a template, and the chloramphenicol resistance gene expression cassette was amplified by PCR using pDONR207 as a template. The primers used were listed in Table S1. Three fragments were used to construct homologous recombinant fragments by fusion PCR.

pCas-SSB, pTargetF targeting the ammonia assimilation-related gene, and donor DNA were introduced into B12 cells by electroporation [30], and the transformation solution was spread on LB plates containing 50 μg/mL chloramphenicol. Transformants were verified by colony PCR. Primers were located in the donor DNA and its downstream in the B12 genome (Table S1).

### 2.9. Pot Trial

Wheat seeds were sterilized as previously described [45] and placed in Petri dishes to air dry in an ultra-clean bench. The air-dried seeds were immersed in a bacterial suspension with OD_600_ = 1.0 for 4 h to swell the seeds sufficiently. Sterile water was used as a control. Excess bacterial suspension was discarded, the seed surface was covered with clean gauze moistened with sterile water, and inoculated at 25 °C for approximately 24 h until the seeds germinated.

Peat soil and vermiculite were evenly mixed in a 1:1 ratio, and 0, 5, and 10 mM NH_4_Cl solutions were added at a w:v ratio of 1. Sprouting seeds were sown into pots at a depth of approximately 1 cm, with about 3 seeds per hole. The pots were then incubated in a greenhouse at 25 °C for 15 d under a 16 h light and 8 h dark cycle. For the first two days, the plants were irrigated with 100 mL of bacterial suspension with an OD600 = 1.0, while the control was irrigated with the same volume of sterile water.

### 2.10. Determination of Wheat Seedling Traits and Rhizosphere Soil Nitrogen Content

After 15 d of growth in pots, the wheat seedlings were removed, and their roots were rinsed with sterile water. The seedlings were then placed in paper bags, heat-killed at 105 °C for 10 min, and kept at 80 °C until constant weight. The dry weight of the seedlings was measured. The total nitrogen content of the wheat seedlings and their rhizosphere soil was measured by the Kjeldahl method, while the ammonia nitrogen content was measured by flow-injection analysis [46]. Seedling nitrogen accumulation (mg seedling^−1^) was calculated by multiplying the nitrogen mass fraction (mg g^−1^) by biomass (g).

### 2.11. Enzyme Activity Analysis

The activities of ammonia assimilation-related enzymes and antioxidant enzymes in wheat seedling roots were analyzed using kits from Solarbio Life Science (Beijing, China). Specifically, the Micro Glutamine Synthetase (GS) Assay Kit was used for GS; the Micro Glutamate Synthase (GOGAT) Assay Kit for GOGAT; the Micro Glutamic Acid Dehydrogenase (GDH) Assay Kit for GDH; the Peroxidase (POD) Activity Assay Kit for POD; the Superoxide Dismutase (SOD) Activity Assay Kit (WST-1 Method) for SOD; and the Catalase (CAT) Activity Assay Kit for CAT. Another set of wheat seedling roots was used to measure dry weight.

### 2.12. Statistical Analysis

All experiments were performed at least three times. Each assay was performed in triplicate on 10 seedlings. Comparisons among the means of three independent experiments used the one-way ANOVA/Tukey’s multiple-comparison test.

## 3. Results

### 3.1. Classification and Identification of Ammonia-Assimilating Strain B12

**Figure 1:**
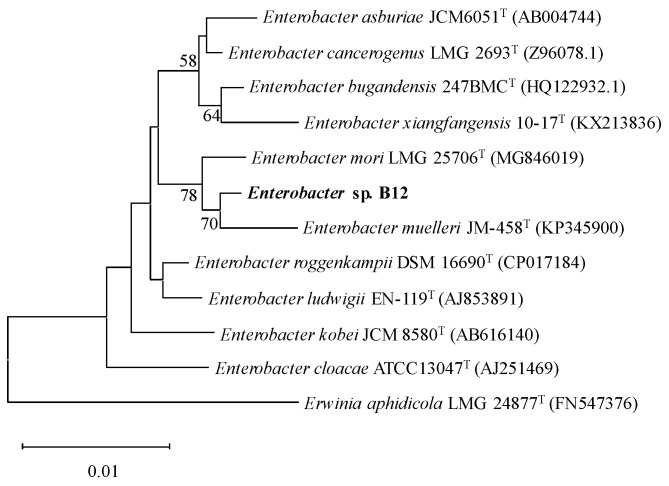
The phylogenetic tree constructed by the neighbor-joining method by MEGA 6.0 software showing the position of the ammonia-assimilating strain B12. Bootstrap values and a scale bar depicting substitution rate per site were indicated. *Erwinia aphidicola* LMG 24877^T^ was also used as an outgroup.

Microscopic observation showed that ammonia-assimilating strain B12 was rod-shaped, and Gram staining was negative. The 16S rRNA sequence of B12 (GenBank: PP301318) was compared with the type strains from the EzBioCloud database, and the phylogenetic tree was constructed using the neighbor-joining method in MEGA 6.0 software (Figure 1). B12 was most closely related to *Enterobacter muelleri* JM-458^T^, with a 16S rRNA sequence similarity of up to 98.80%, suggesting that B12 may belong to *Enterobacter muelleri* and was thus designated as *Enterobacter* sp. B12.

### 3.2. Ammonia Assimilation Rate and Ammonia Tolerance of Enterobacter sp. B12

**Figure 2:**
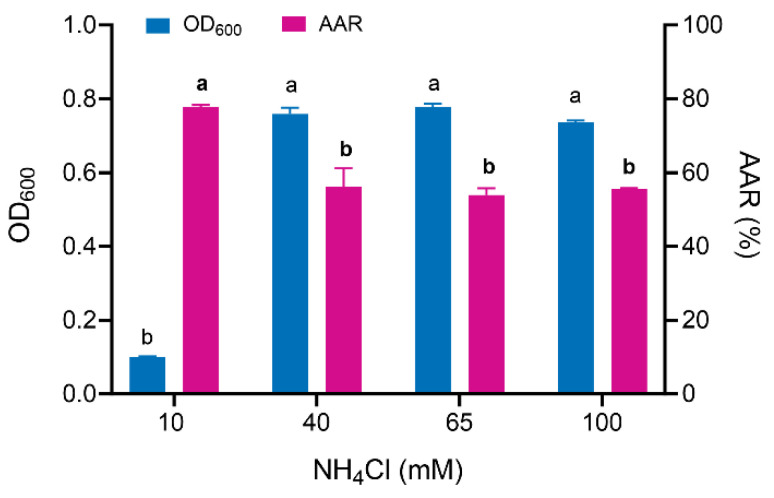
Effect of ammonia concentration on the growth and ammonia-assimilation rate of ammonia-assimilating strain B12. Data are means and standard deviations (SDs) of three biological replicates, each conducted in triplicate. AAR, ammonia assimilation rate. Bars of OD_600_ or AAR marked with different lowercase letters were significantly different at *p* < 0.05 based on one-way ANOVA/Tukey’s multiple-comparison test.

The growth of OD_600_ and ammonia assimilation rate of *Enterobacter* sp. B12 were determined by shake flask incubation for 24 h in an ammonia–nitrogen medium containing 10 mM–100 mM NH_4_Cl. At an NH_4_Cl concentration of 10 mM, the bacterial mass was approximately 13% of that observed at NH_4_Cl concentrations of 40 mM to 100 mM, with an ammonia assimilation rate of 77.85%. No differences were observed in the bacterial mass and ammonia assimilation rates between NH_4_Cl concentrations of 40 mM and 100 mM, with ammonia assimilation rates ranging from 53.88% to 56.29% (Figure 2), indicating that B12 exhibited high ammonia tolerance.

### 3.3. Growth of Enterobacter sp. B12 in Relation to Ammonia Assimilation

**Figure 3:**
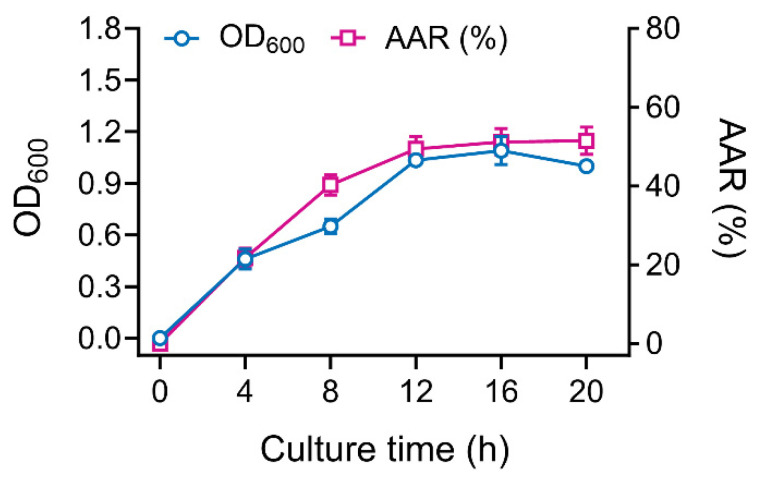
Growth curve and ammonia assimilation rate curve of *Enterobacter* sp. B12 grown on ammonia–nitrogen medium containing 65 mM NH_4_Cl. Data are means and SD of three biological replicates, each conducted in triplicate.

*Enterobacter* sp. B12 was cultured in shaking flasks in ammonia–nitrogen medium with an NH_4_Cl concentration of 65 mM, and the growth curve and ammonia assimilation rate curve were plotted, which were essentially superimposed (Figure 3), and the bacterial mass and the ammonia assimilation rate showed a positive correlation (*p* < 0.001). No nitrite or nitrate was produced during the growth process, indicating that ammonia was assimilated for bacterial growth.

### 3.4. Effects of Ammonia and Glutamine on Enterobacter sp. B12 Growth

**Figure 4:**
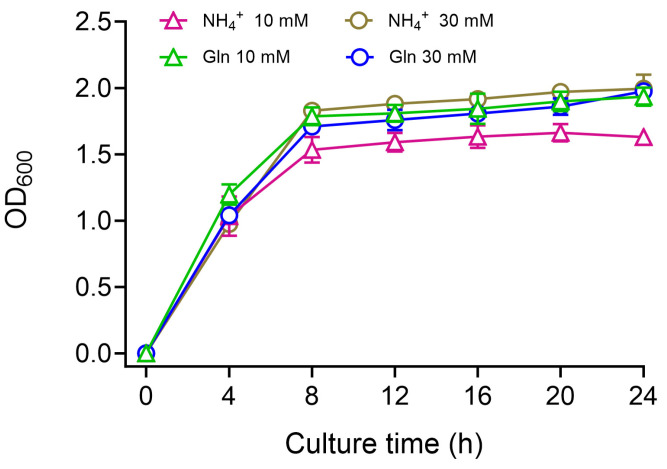
Growth curve of *Enterobacter* sp. B12 on ammonia or glutamine as the sole nitrogen source. Data are means and SD of three biological replicates, each conducted in triplicate.

*Enterobacter* sp. B12 was cultured in ammonia–nitrogen medium in shaking flasks with 10 mM and 30 mM NH_4_Cl or glutamine as the sole nitrogen source, respectively, and its growth curve was plotted. At the NH_4_Cl concentration of 10 mM, the growth rate did not differ from that of 10 mM glutamine in the first 4 h, and the growth rate and growth of the bacterial mass were lower than that of 10 mM glutamine after 4 h (Figure 3). When the concentration of NH_4_Cl and the concentration of glutamine were both 30 mM, there was no difference in the growth rate and bacterial mass (Figure 4). This indicates that both ammonia and glutamine are preferred nitrogen sources for B12.

### 3.5. Whole Genome Sequencing and Identification of Ammonia Assimilation-Related Genes in Enterobacter sp. B12

**Figure 5:**
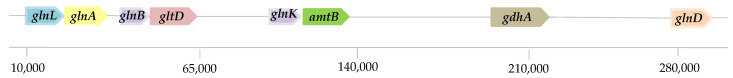
Distribution of genes related to ammonia assimilation in the genome of *Enterobacter* sp. B12.

The complete genome sequence of *Enterobacter* sp. B12 was analyzed using the Illumina HiSeq 2000 sequencing platform and deposited in GenBank under the accession numbers PRJNA1135260. The circular chromosome genome length was 4,506,957 bp with 56.05% GC content. It contained 4335 protein-coding genes, and the length of the protein-coding region was 403,671,714, accounting for 89.57% of the total length. There were 86 RNA genes, of which 79 were tRNA genes and 7 were rRNA genes. Ammonia assimilation-related genes annotated in the genome included one GDH gene (*gdhA*), one GS gene (*glnA*), one GOGAT gene (*gltD*), one ammonia transporter protein gene (*amtB*), and several ammonia assimilation-regulated genes *glnL*, *glnB*, *glnD*, and *glnK* (Figure 5). No nitrification-related genes were annotated in the genome.

**Figure 6:**
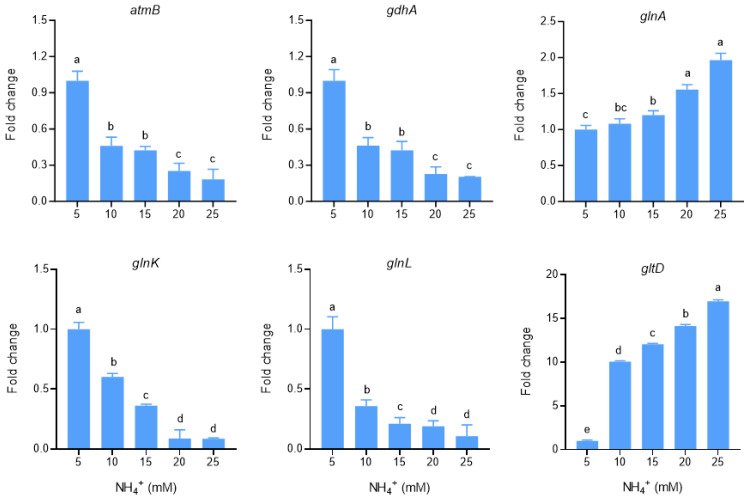
Effect of ammonia concentration on the expression of genes related to ammonia assimilation in *Enterobacter* sp. B12. Data are means and SD of three biological replicates, each conducted in triplicate. Bars marked with different lowercase letters were significantly different at *p* < 0.05 based on one-way ANOVA/Tukey’s multiple-comparison test.

Quantitative PCR analysis showed that *gdhA*, *GlnK*, *GlnL*, and *amtB* were highly expressed under high-carbon and low-ammonia conditions (5 mM), whereas *glnA* and *gltD* were highly expressed under high-carbon and high-ammonia conditions (10–25 mM) (Figure 6).

### 3.6. Effects of Mutations in Genes Related to Ammonia Assimilation on the Growth of Enterobacter sp. B12

To investigate the effects of ammonia assimilation genes on ammonia assimilation and growth of *Enterobacter* sp. B12, mutant strains B12Δ*gdhA*, B12Δ*glnA*, B12Δ*gltD*, and B12Δ*amtB* were constructed (Figure S1).

**Figure 7:**
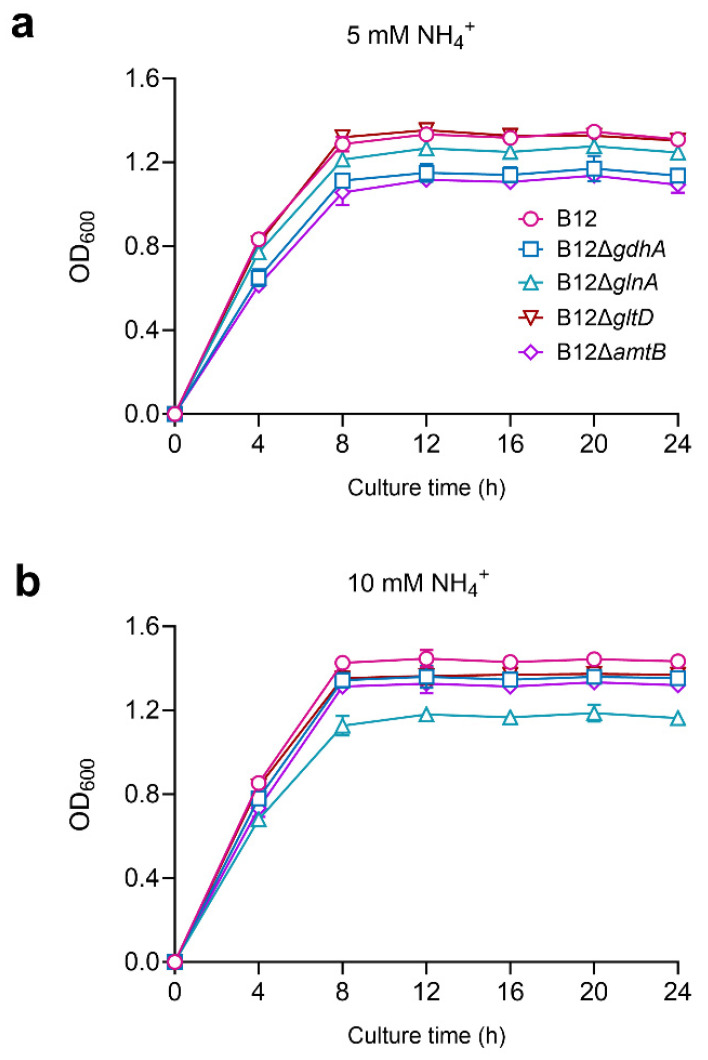
Growth curves of *Enterobacter* sp. B12 and derived mutant strains cultured under different ammonium concentrations. (**a**) 5 mM NH_4_^+^ in ammonia–nitrogen medium; (**b**) 10 mM NH_4_^+^ in ammonia–nitrogen medium. Data are means and SD of three biological replicates, each conducted in triplicate.

When grown on ammonia–nitrogen medium under high-carbon (20 mM) and low-ammonia (5 mM) conditions, the growth rate and biomass (OD_600_) of B12Δ*gdhA* and B12Δ*amtB* were lower than those of the WT and the other mutant strains (Figure 7a). Under high carbon and high-ammonia conditions (10 mM), only B12Δ*glnA* exhibited a lower growth rate and biomass compared to the WT and other mutants (Figure 7b), suggesting that the GDH pathway and amtB are crucial for ammonia assimilation under high-carbon and low-ammonia conditions, while GS and amtB are essential for ammonia assimilation under high-carbon and high-ammonia conditions.

### 3.7. Effects of Enterobacter sp. B12 on Wheat Growth, Ammonia Nitrogen, and Total Nitrogen Accumulation

**Figure 8:**
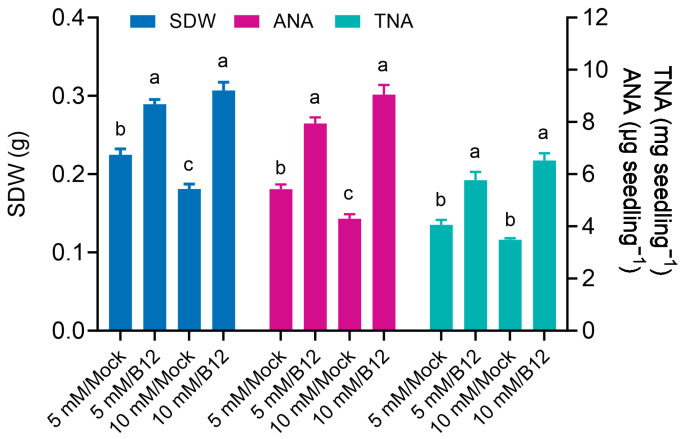
*Enterobacter* sp. B12 promoted wheat seedling growth and nitrogen utilization. SDW, seedling dry weight; ANA, ammonia N accumulation; TNA, total N accumulation. Bars represent the mean values ± standard deviation (SD) with three replications and ten seedlings in each replicate. Same color bars marked with different lowercase letters were significantly different at *p* < 0.05 based on one-way ANOVA/Tukey’s multiple-comparison test.

Wheat seedlings grown in pots for 15 days under conditions of 5 mM and 10 mM ammonia exhibited enhanced growth when inoculated with B12, with dry weights increasing by 28.82% and 69.41%, respectively, compared to the uninoculated mock seedlings. The dry weight of mock seedlings under 10 mM ammonia was lower than that under 5 mM, which should be the toxic effect of high ammonia. Additionally, B12 inoculation improved ammonia N accumulation in the seedlings, with increases of 46.31% and 1.11-fold relative to the mock seedlings under 5 mM and 10 mM ammonia, respectively. Similarly, total N accumulation in seedlings increased by 42.21% and 87.31% under the same conditions, respectively, when inoculated with B12 (Figure 8).

### 3.8. Effects of Enterobacter sp. B12 on Ammonia N and Total N Contents in Wheat Seedling Rhizosphere Soil

**Figure 9:**
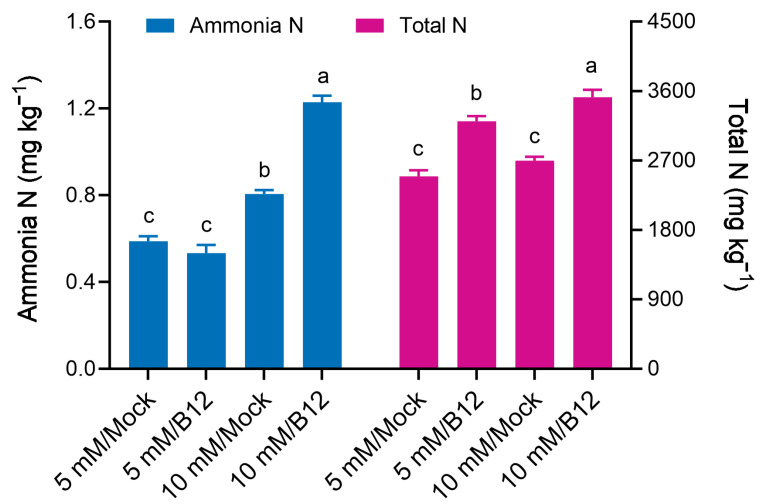
Ammonia N and total N content in the rhizosphere soil of wheat seedlings inoculated with and without *Enterobacter* sp. B12. Bars represent mean ± SD with three replications and ten seedlings in each replicate. Same color bars marked with different lowercase letters were significantly different at *p* < 0.05 based on one-way ANOVA/Tukey’s multiple-comparison test.

Potted wheat inoculated with *Enterobacter* sp. B12 showed no difference in the ammonia N content of the seedling rhizosphere soil compared to mock treatment under 5 mM ammonia conditions. However, the total N content increased by 28.55% compared to the mock treatment. Under 10 mM ammonia conditions, inoculation with B12 increased seedling rhizosphere soil ammonia N content and total N content by 52.48% and 30.59%, respectively, compared to mock treatment (Figure 9). This indicated that inoculation with ammonia-assimilating bacteria improved the immobilization of ammonia and total N in the rhizosphere soil.

### 3.9. Effects of Enterobacter sp. B12 on Ammonia Assimilation and ROS-Scavenging Enzyme Activities in Wheat Seedling Roots

**Figure 10:**
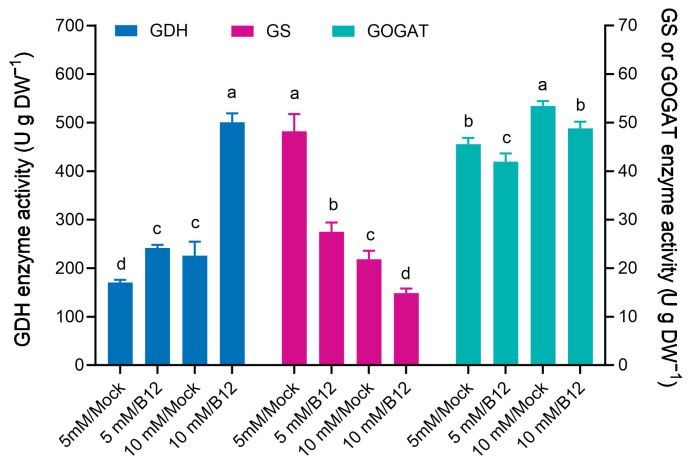
Ammonia assimilation enzyme activities in wheat seedling roots inoculated with and without *Enterobacter* sp. B12. Bars represent mean ± SD with three replications and ten seedlings in each replicate. Same color bars marked with different lowercase letters were significantly different at *p* < 0.05 based on one-way ANOVA/Tukey’s multiple-comparison test.

Potted wheat roots at 5 mM and 10 mM inoculated with *Enterobacter* sp. B12 showed a 41.72% and 1.22-fold increase in GDH enzyme activity compared to mock roots, respectively. Conversely, GS enzyme activity was reduced by 42.95% and 31.93%, and GOGAT enzyme activity was reduced by 7.89% and 8.68% compared to mock roots, respectively (Figure 10).

**Figure 11:**
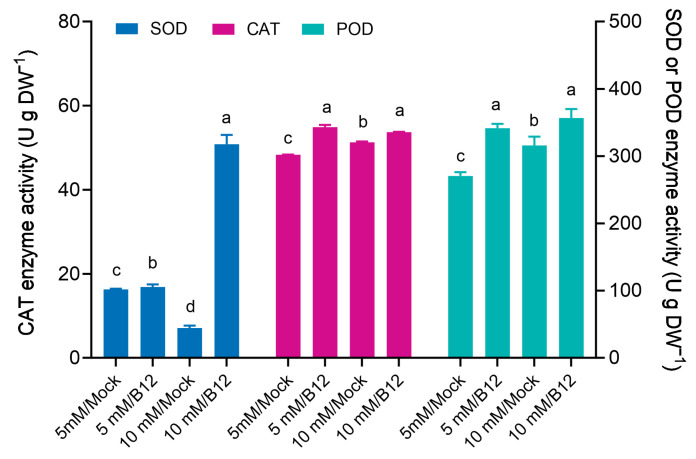
ROS-scavenging enzyme activities in wheat seedling roots inoculated with and without *Enterobacter* sp. B12. Bars represent mean ± SD with three replications and ten seedlings in each replicate. CAT, catalase; POD, peroxidase; SOD, superoxide dismutase. Same color bars marked with different lowercase letters were significantly different at *p* < 0.05 based on one-way ANOVA/Tukey’s multiple-comparison test.

The SOD enzyme activity of mock wheat roots was lower under 10 mM ammonia than under 5 mM ammonia, indicating that high ammonia induces oxidative stress in wheat roots. The activities of the antioxidant enzymes SOD, CAT, and POD were increased in wheat roots inoculated with B12 under 5 mM and 10 mM ammonia (Figure 11).

### 3.10. Effects of Enterobacter sp. B12 on the Expression of Genes Related to Ammonia Assimilation and Genes for Ammonia and Amino Acid Transporter in Wheat Seedling Roots

**Figure 12:**
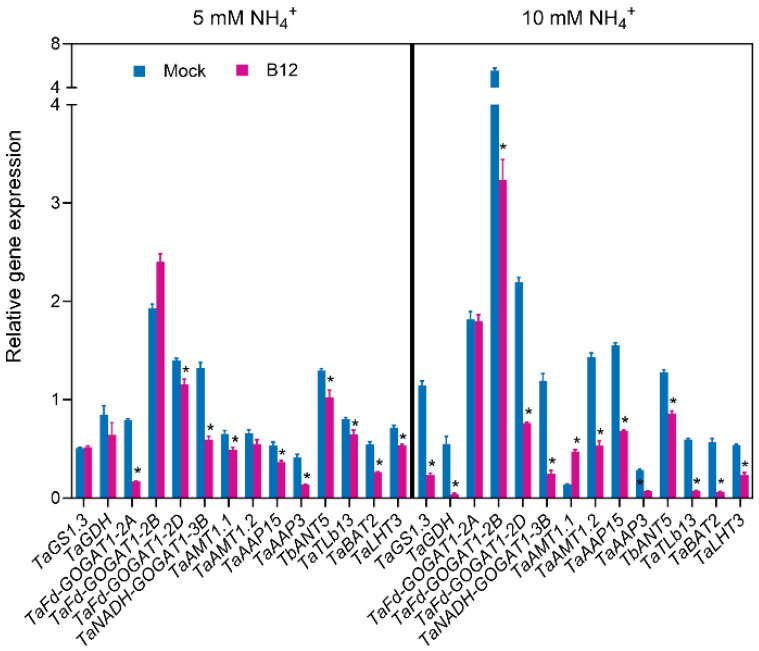
Relative expression of genes related to ammonia assimilation and genes for ammonia and amino acid transporters in wheat seedling roots inoculated with and without *Enterobacter* sp. B12. Bars represent mean ± SD with three replications and ten seedlings in each replicate. Details of genes were provided in Table S2. Bars marked with an asterisk showed significantly different results at *p* < 0.05 between those inoculated with *Enterobacter* sp. B12 and the mock treatment based on a paired *t*-test.

The expression levels of genes related to ammonia assimilation, as well as ammonia and amino acid transporter genes, in *Enterobacter* sp. B12-inoculated wheat roots treated with 5 mM ammonia were lower than those of the mock roots, except for *TaGS1.3*, *TaGDH*, *TaFd-GOGAT1-2B*, and *TaAMT1.2*, which showed no significant difference. When treated with 10 mM ammonia, the expression levels of these genes in B12-inoculated wheat roots were also lower compared to the mock roots, except for *TaFd-GOGAT1-2A*, which showed no difference, and *TaAMT1.1*, whose expression was increased relative to the mock roots (Figure 12).

## 4. Discussion

*Enterobacter* sp. B12 exhibited typical characteristics of ammonia-assimilating bacteria, with high ammonia assimilation capacity and ammonia tolerance. It does not nitrify ammonia, and both glutamine and ammonia serve as optimal nitrogen sources (Figure 1, Figure 2 and Figure 3) [47,48]. B12 also has two ammonia assimilation pathways, assimilating ammonia via the GDH pathway at low-ammonia levels and the GS-GOGAT pathway at high-ammonia levels (Figure 6 and Figure 7). The regulation of the ammonia assimilation pathway by ammonia concentration differed from that of *E. coli* and *Bacteroides thetaiotaomicron*. In humans, colonic bacterium *Bacteroides thetaiotaomicron*, *gdhA*, *gltD*, *glnK*, and *amtB* were highly expressed at high-carbon and low-ammonia conditions, and *gdhB* and *glnA* were highly expressed at high-carbon and high-ammonia conditions [12,47,48].

In the global nitrogen cycle, the highest nitrogen fluxes are observed in the conversion of ammonia to organic nitrogen [47,48,49]. Bacterial ammonia assimilation plays an important role in livestock production and composting. Assimilated ammonium accounts for up to 70% of microbial protein production in the rumen, meeting up to 85% of the protein requirements of the ruminant host [12,47,48]. *Bacillus velezensis* LG37 efficiently purifies aquaculture water and improves tolerance to ammonia toxicity in grass carp [47,48,50]. Bacterial assimilation of ammonia to produce bioavailable nitrogen and humus is a critical pathway for nitrogen immobilization during composting [13,47,48,51,52]. Agriculture is equally challenged with preserving ammonia to reduce nitrogen loss. Enhanced efficiency fertilizers (EEFs) and membrane-/film-based mulching methods can reduce ammonia volatilization and nitrogen losses, but the performance of EEFs varies across agroecological locations due to differences in soil texture, drainage, organic matter, pH, slope, temperature, and rainfall [53]. In addition, most EEFs are currently more expensive than straight urea [3]. Membrane-/film-based mulching methods have problems such as unstable film formation, easy breakage, high cost, and easy aggregation [54]. The use of ammonia-assimilating biofertilizers could be an effective measure to reduce ammonia volatilization and nitrogen losses in crop fields.

In this study, inoculation of potted wheat with the ammonia-assimilating bacterium *Enterobacter* sp. B12 increased seedling dry weight, ammonia nitrogen, and total nitrogen accumulation (Figure 8), rhizosphere soil ammonia nitrogen and total nitrogen content (Figure 9), and root enzyme activities (SOD, CAT, and POD) (Figure 11). This showed that B12 promoted N absorption by wheat, improved soil immobilization of ammonia nitrogen and total nitrogen, attenuated the stress effects of low or high ammonia by either increasing the ammonium concentration in the root microenvironment by increasing the root uptake area under low-ammonium conditions or reducing it by ammonia assimilation under high-ammonium conditions, and improved antioxidant enzyme activities in wheat roots. In addition, except for GDH enzyme activity, which was higher than that of mock roots, GS and GOGAT enzyme activities were lower (Figure 10), and the expression of ammonia assimilation-related genes, including GS, GDH, GOGAT, ammonia transporter protein genes, and amino acid transporter protein genes, was lower than that of mock roots (Figure 12). It was suggested that ammonia-assimilating bacteria were able to modulate the expression of genes related to ammonia and nitrogen metabolism in wheat roots.

Several previous studies showed that plant growth-promoting rhizobacteria (PGPR) *Bacillus* mixtures and *Achromobacter* are able to increase the expression of genes related to the transportation and assimilation of N compounds in roots [47,48,55,56]. Obviously, B12 altered the expression of genes related to the assimilation and transport of N in wheat roots differently from these PGPRs but similarly to mycorrhizal fungi. The arbuscular mycorrhizal fungus (AMF) *Glomus intraradices* significantly increases glutamine synthetase and glutamate dehydrogenase activities in carrot roots [47,48,57]. AMFs downregulate ZmAMT1;1a and ZmAMT1;3 protein abundance and transport activities expressed in the root epidermis, suggesting a trade-off between mycorrhizal and direct root N uptake pathways [47,48,58]. AMFs elevate NH_4_^+^ concentration, GS and GOGAT activities in *Populus cathayana*. Additionally, the transcriptional levels of NH_4_^+^ transporter genes (*PcAMT1-4* and *PcAMT2-1*) were significantly downregulated in *P. cathayana* roots [47,48,59]. There is an exchange of NH_4_^+^ and NO_3_^−^ between mycorrhizal fungi and plants [60] and possibly amino acids [61,62]. NH_4_^+^ and amino acid exchange may occur between B12 and wheat.

Plant–PGPR form a ‘love match’—plant roots secrete approximately 5–20% of total photosynthetically fixed carbon to feed off the PGPR and provide a place for it to live, and in return PGPR promotes plant growth through various mechanisms [63]. PGPR promote plant growth through both direct and indirect mechanisms, including nitrogen fixation, phosphate solubilization, potassium solubilization, siderophore production, and alteration of plant hormone levels, which enhance root surface area and morphology, thereby increasing nutrient absorption. They also increase plant resistance to biotic and abiotic stresses [47,48,64,65,66]. However, PGPR strains that reduce nitrogen loss through ammonia assimilation, enhance plant ammonia tolerance, and modulate the expression of plant nitrogen metabolism-related genes have not been reported. Therefore, ammonia-assimilating bacteria should be a new type of PGPR. Ammonia-assimilating bacteria, along with nitrogen-fixing bacteria, are probably the PGPRs that provide the most nitrogen to plants.

## 5. Conclusions

The ammonia-assimilating strain isolated from mushroom compost was identified as *Enterobacter* sp. B12, which had high ammonia-assimilating ability and high ammonia tolerance and assimilated ammonia via the GDH pathway at low-ammonia levels and the GS-GOGAT pathway at high-ammonia levels. Inoculation of potted wheat with this strain increased seedling dry weight, nitrogen accumulation, rhizosphere soil nitrogen content, and root enzyme activities, including GDH, SOD, CAT, and POD, while downregulating the expression of GS, GDH, GOGAT, ammonia transporter protein genes, and amino acid transporter protein genes in wheat roots. These mechanisms of plant growth promotion are different from those reported for PGPR. Therefore, ammonia-assimilating bacteria should be a new type of PGPR.

## Data Availability

The raw sequence data are available at the NCBI Sequence Read Archive, under Bioproject number PRJNA1135260.

## Supplementary Materials

The following supporting information can be downloaded at: https://www.mdpi.com/article/10.3390/microorganisms13010043/s1. Figure S1: PCR verification of *Enterobacter* sp. B12-derived mutant strains B12Δ*gdhA*, B12Δ*glnA*, B12Δ*gltD*, and B12Δ*amtB*. Table S1: Primers used in this study; Table S2: Primers used for quantitative PCR in this study.
