# Supplementary material for: Ammonia-Assimilating Bacteria Promote Wheat (Triticum aestivum) Growth and Nitrogen Utilization
Source: Microorganisms. 2024 Dec 30;13(1):43. doi: 10.3390/microorganisms13010043 (PMC11767723; doi:10.3390/microorganisms13010043)
Supplement: Supplementary file 1 [file microorganisms-13-00043-s001.zip › microorganisms-3387622-supplementary.pdf]

Supplementary Information for

Ammonia-assimilating bacteria promote wheat (*Triticum aestivum*) growth and nitrogen utilization

Yuqian Gao, Qi Zhang, Yuannan Chen, Yanqing Yang, Chenxiao Zhou, Jiayang Yu, Yanan Li*, Liyou Qiu*

*Corresponding author

liyanan@henau.edu.cn, qliyou@henau.edu.cn

College of Life Sciences, Henan Agricultural University

218 Ping'an Avenue, Zhengzhou 450046, China

Table S1 Primers used in this study

Name	Nucleotide sequence (5′–3′)	Trials
P1	CGGGATCCAGAGTTTGATCCTGGCTCAGAACGAACGCT	Strain identification
P6	CGGGATCCTACGGCTACCTTGTTACGACTTCACCCC	
sgRNA-*gdhA*-F	ACAGCGCATGCGTCTTTACGTTTTAGAGCTAGAAATAGCAAGTTAAAATAA	Insertion of N_{20} sequence
sgRNA-*gdhA*-R	TAAAGACGCATGCGCTGTTACTAGTATTATACCTAGGACTGAGCTAGCTG	
sgRNA-*glnA*-F	AGCAGGCGCTGTATTACATGTTTTAGAGCTAGAAATAGCAAGTTAAAATAA	
sgRNA-*glnA*-R	TGTAATACAGCGCCTGCTCACTAGTATTATACCTAGGACTGAGCTAGCTG	
sgRNA-*gltD*-F	AAACCGAACGCCATCACCAGTTTTAGAGCTAGAAATAGCAAGTTAAAATAA	
sgRNA-*gltD*-R	GGTGATGGCGTTCGGTTTCACTAGTATTATACCTAGGACTGAGCTAGCTG	
sgRNA-amtB-F	ATCAGCCCGACGGTAATACGTTTTAGAGCTAGAAATAGCAAGTTAAAATAA	
sgRNA-*amtB*-R	TATTACCGTCGGGCTGATTACTAGTATTATACCTAGGACTGAGCTAGCTG	
gdhA-UP-F	CTGCACCGTTTGATTCTG	Amplification of the upstream homology arm of the *gdhA* gene
gdhA-UP-R	CTCTGGTTTACCGTTATAGTC	
gdhA-DOWN-F	CTGCCAACTTAGTCGACTACCATCCCTCTGAACGTTTTC	Amplification of the downstream homology arm of the *gdhA* gene
gdhA-DOWN-R	GGTTTGTGCAGGATAAAGATC	
gdhA-CmR-F	GACTATAACGGTAAACCAGAGCCTTGAGCAACTGATAGCTG	Amplification of the *CmR* gene
gdhA-CmR-R	GTAGTCGACTAAGTTGGCAG	
glnA-UP-F	CATCGACCAGCAATATGC	Amplification of the upstream homology arm of the *glnA* gene
glnA-UP-R	GTTTTACCCGGCGTGATAAG	
glnA-DOWN-F	CTGCCAACTTAGTCGACTACCTCCAGATTGGTCATTGTC	Amplification of the downstream homology arm of the *glnA* gene
glnA -DOWN-R	GGTTTGTGCAGGATAAAGATC	
glnA-CmR-F	CTTATCACGCCGGGTAAAACCCTTGAGCAACTGATAGCTG	Amplification of the *CmR* gene
glnA-CmR-R	GTAGTCGACTAAGTTGGCAG	
YZ-*glnA*-F	GTGAACACTATCCCATATCAC	PCR validation of knockout strain
YZ-*glnA*-R	GCTTTGTGATCGCTTTCAC	
gltD-UP-F	GATGAAGTGCGCTTCAAC	Amplification of the upstream homology arm of the *gltD*gene
gltD-UP-R	GCCTGTTTTTATTTCACGAC	
gltD-DOWN-F	CTGCCAACTTAGTCGACTACCAACAGGGCTTTAACATCG	Amplification of the downstream homology arm of the *gltD* gene
gltD-DOWN-R	CGTATGTCCTGGATGAAGAC	

gltD-CmR-F	GTCGTGAAATAAAAACAGGCCCTTGAGCAACTGATAGCTG	Amplification of the *CmR* gene
gltD-CmR-R	GTAGTCGACTAAGTTGGCAG	
YZ-*gltD*-F	GTGAACACTATCCCATATCAC	PCR validation of knockout strain
YZ-*gltD*-R	GAAGTGCTGGACGTTGATAC	
amtB-UP-F	CATGCCGAGCTTTATCGC	Amplification of the upstream homology arm of the *amtB* gene
amtB-UP-R	GTACGGATGCGAATGACG	
amtB-DOWN-F	CTGCCAACTTAGTCGACTACCGGTTGCCGTTTTTAGTG	Amplification of the downstream homology arm of the *gltB* gene
amtB-DOWN-R	GTCCGTTCAACATGAATGAG	
amtB-CmR-F	CGTCATTCGCATCCGTACCCTTGAGCAACTGATAGCTG	Amplification of the *CmR* gene
amtB-CmR-R	GTAGTCGACTAAGTTGGCAG	
YZ-*amtB*-F	GTGAACACTATCCCATATCAC	PCR validation of knockout strain
YZ-*amtB*-R	GGAGTTTTATACTCAGGATGG	

Table S2 Primers used for quantitative PCR in this study

Genes	Forward primers (5′–3′)	Reverse primers (5′–3′)
rpoB	GCCAAGCCGATTTCTGGAGCA	CGTTTCGATTGGACATACG
gdhA	TGTGAAATCAAAGCCAGCC	CACGCCGTTGCTAATCAA
glnA	CCAACCACCAACTCCTACAAG	CGGGATACGGATAGAAGCAG
gltD	ATTGCCAACACCAAGCAG	AACTGGAACTCTACGCCCTC
amtB	GCAATGCGTTCTTTGGTAAC	TAGGCACATAGGAGAGCGTC
TaActin	TTGCTGACCGTATGAGCAAG	ACCCTCCAATCCAGACACTG
TaAAP3	GTACAGCCAGCCGATCTACC	CACCTTCCGCTGGATGAAGT
TaAAP7	TCTGGCCGTTGTGTGTCTAC	ATGATACCGACGATGGACGC
TaAAP15	CTCAGCATGACCACCGTCTT	AGCCAGTAGGGCTCGTAGAA
TaAAP17	AATGGAGTCATCAAGGGCGG	CTCAGCGTGTCCTCGATCTC
TaAAP18	CTGCTTGCGGATTGTTACCG	TTAACCTTACGCACCGCACT
TaATLb13	AGTGGCATGCCCTTTGCTAT	GCGACAGAGTCTTGTCACCA
TaLHT3	ACCAGATACCTCCCAACGGA	GGCATCGCGTAGATCTGGAA
TaLHT8	CTGTCCTACTCGACCATCGC	TCCACATGGGCTTCTTGGAC
TaANT5	GATGGCGGCAACTACACCTA	GGCCGATGAAGACGAGGTAG
TaBAT2	GGAGACCAAGAACGCAGACA	CGCAGAAGTAAATGGCGACG
TaAMT1.1	TGGAGTCCTCGTCGGTGTAG	TCATCTGCGGGTTCGTGT
TaAMT1.2	TGAGGTAGGCGACGAACTGC	CAACGGGTTTATCGGGAAGC
TaNDAH-GOGAT1-3A	TGGCGAAAGATGGAGTGGA	TTTAAGTGCAAGGCGGTTG
TaNADH-GOGAT1-3B	GTGAATGCTAATGTAGGGAGTG	TTTGGTATTTGCGTGGAGA
TaNADH-GOGAT1-3D	GGGAGCAGCCTTCTCGTAT	TTAGCACCCTGAGCCATTT
TaFd-GOGAT1-2A	CAGGGAAACTTGAACTGGAT	TTTACTGATAATGTCGGATGGT
TaFd-GOGAT1-2B	AGGTTAAGCGTTCTTACTGCG	AAGCGTCAGCGAACCAATT
TaFd-GOGAT1-2D	TTCTGAGCGAATGGGAGG	TTTGGCGATTGGGCTTT
TaGS1.3	GAAGAAGAAGAAGAGGTAGCCATG	AACAGAACCCATCAAAGCCAC
TaGDH	GGCACCAACGCACAGACA	GCCAACATTACCGAATCCCT

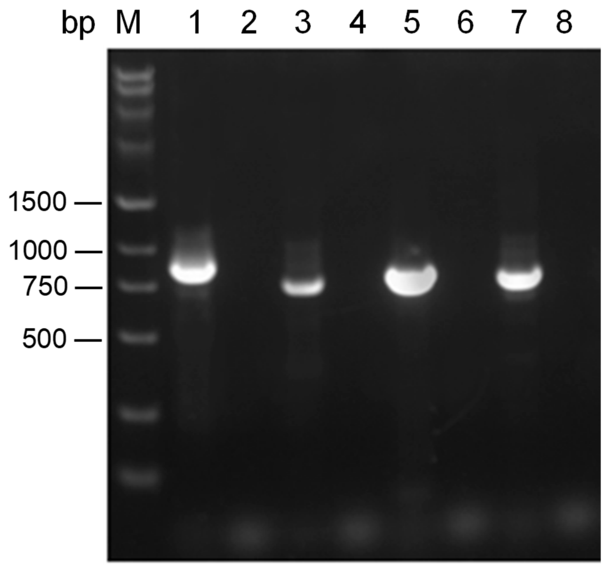

Figure S1 PCR verification of *Enterobacter* sp. B12 derived mutant strains B12Δ*gdhA*, B12Δ*glnA*, B12Δ*gltD* and B12Δ*amtB*. M: Super DNA marker Zm108; lane 1, 3, 5, 7: B12Δ*gdhA*, B12Δ*glnA*, B12Δ*amtB*, B12Δ*gltD*, amplified bands of homologous recombination knockout fragments; lane 2, 4, 6, 8: WT, no amplified bands of homologous recombination knockout fragments for deletion of *gdhA*, *glnA*, *amtB*, *gltD*.
